# Assessing the feasibility and impact of an adapted resistance training intervention, aimed at improving the multi-dimensional health and functional capacity of frail older adults in residential care settings: protocol for a feasibility study

**DOI:** 10.1186/s40814-019-0470-1

**Published:** 2019-07-05

**Authors:** Paul Doody, Janet M. Lord, Anna C. Whittaker

**Affiliations:** 1 School of Sport Exercise and Rehabilitation Sciences (G35), University of Birmingham, Birmingham, UK; 2 MRC-ARUK Centre for Musculoskeletal Ageing Research, Institute of Inflammation and Ageing, University of Birmingham, Birmingham, UK; 3 NIHR Birmingham Biomedical Research Centre, University Hospital Birmingham, Birmingham, UK; 4 Faculty of Health Sciences and Sport, University of Stirling, Sterling, UK

**Keywords:** Care home, Elderly, Exercise, Feasibility study, Frail, Functional capacity, Geriatric, Health, Intervention, Older adults, Physical activity, Residential care

## Abstract

**Background:**

Frailty is a common and clinically significant condition in older adults, predominantly due to its association with adverse health outcomes such as hospitalisation, disability and mortality. Exercise interventions have been shown to be a beneficial treatment for frail older adults. However, more high-quality studies are needed within this area to assess the feasibility and impact of these interventions in frail geriatric populations within different settings, and with regards to their impact on broader aspects of health and wellbeing.

**Methods:**

This study will utilise an interventional, randomised, controlled research design in order to assess the feasibility (acceptability, demand, implementation, practicality, adaptation, integration, expansion) and potential impact (limited-efficacy testing) of a specially adapted resistance training intervention; aimed at improving the multi-dimensional health and functional capacity of frail geriatric care home residents.

**Discussion:**

The most immediate implication of this research from a scientific perspective is informing the feasibility, and potential efficacy, of a proposed future clinical trial within this setting. Additionally, if the study proves feasible, and the limited-efficacy testing proves positive, this study also has the potential to lead to advancement in the care for frail geriatric populations within residential care settings; and the ability to measurably improve various aspects of health and functional capacity within this population. This study has been granted a favourable ethical opinion by the London Harrow NHS Research Ethics Committee and is sponsored by the University of Birmingham. The findings of this study will be disseminated through publication in open access scientific journals, public engagement events, online via social media, conference presentations and directly to study participants.

**Trial registration:**

ClinicalTrials.gov: NCT03141879. Registered 5 May 2017.

**Electronic supplementary material:**

The online version of this article (10.1186/s40814-019-0470-1) contains supplementary material, which is available to authorized users.

## Background

Frailty is a common and clinically significant condition within geriatric populations [1]; the latter predominantly due to its association with adverse health outcomes such as hospitalisation, disability and mortality [1–6]. The exact prevalence of frailty within this population is poorly defined due to the lack of a single standardised operational definition for the classification of frailty. However, it is generally believed that the prevalence of frailty amongst community-dwelling older adults ranges between 7.0 and 16.3% [1, 7]. Presently, there are no well-evidenced, pooled estimates of the prevalence of frailty amongst older adults in care home settings. Although, it could be postulated that this prevalence would likely be higher than that of community-dwelling older adults, given that older adults living in care homes typically tend to be chronologically older, and often exhibit a greater number of comorbidities and a reduced functional capacity. However, these differences routinely become non-significant once standardised for age [8]. Additionally, the estimated prevalence of frailty in nursing homes (where qualified nursing care is required, in addition to care assistance) is approximately 52.3% [9]. As such, the prevalence of frailty in care homes likely lies somewhere in between that of community-dwelling older adults and nursing home residents; given the inherent nature of these respective settings, and the demographics of the individuals who occupy them.

Although there is no one standardised and universally utilised operational definition for the classification of frailty, one of the most commonly utilised is the Fried frailty phenotype [2]. This proposes that frailty be defined as a clinical syndrome in which three or more of the five following criteria are present: unintentional weight loss (≥ 10 lbs in the past year), self-reported exhaustion, weakness (grip strength), slow walking speed and low levels of physical activity.

Exercise interventions have been proposed as potentially offering the best form of treatment for frail older adults [10]; with exercise shown to be a significantly beneficial treatment for frail older adults [11–13] and even shown to mediate the reversal of frailty in some cases [12, 14] (i.e. moving from a state classified as frail, to pre-frail or pre-frail to robust [2]. However, while there is evidence of the benefits of exercise relating to the prevention, treatment, and reversal of frailty, it is universally noted that there needs to be more high-quality studies within this area to truly assess the impact of exercise in frail geriatric populations, particularly relating to its effects on broader aspects of health and well-being [1]. This present study will assess the feasibility and potential efficacy of a specialised exercise intervention, in the form of a 12-week, 3 to 4 days per week, resistance training programme for frail older adults within a residential care setting. Although the proposed future clinical trial which this feasibility study will inform will be 24 weeks in duration (12 weeks of intervention, with a 12 week follow-up), this present feasibility study will be 12 weeks in duration (6 weeks of intervention, with a 6-week follow-up), as it was determined that the full 24 weeks of the proposed future clinical trial will not be necessary to determine its feasibility. Feasibility will relate to the eight main areas of focus for feasibility studies [15], while potential efficacy will be assessed through the limited-efficacy testing of the impact of the intervention on the patient-centred outcomes relating to multi-dimensional health and functional capacity.

Such research is very timely and pertinent, as current demographic trends indicate that by the year 2030 almost one in six of the European population will be aged 60 or over, and the number of older people will grow to 247 million by 2050, representing a 35% increase from 2017, with one in four older adults being above 85 by 2040 [16]. This, coupled with continual progressive declines in the rate of physical activity, not only in older adults but at all stages of the lifespan [17], leaves the population particularly susceptible to the development of disease and comorbidities associated with a lack of physical activity and an increase in sedentary behaviour [18]. Therefore, there is an urgent need to examine the effect of such interventions within this setting, and whether these interventions can be employed to improve various aspects of health in frail older populations in assisted living facilities, as well as their efficacy in specifically treating, preventing and reversing frailty.

## Methods

### Aims and objectives

The primary aim of this study is to assess the feasibility of a proposed future clinical trial in this setting, which aims to assess the impact of a specially adapted resistance training intervention on the physiological, psychological, cognitive, social and emotional health and functional capacity of frail geriatric populations within a residential care setting; recognising health as a multi-factorial concept incorporating multiple inter-related dimensions. The secondary aim of this feasibility study is to assess the potential efficacy of the intervention on the primary dependent variables of the proposed future clinical trial within this setting.

The primary and secondary aims of this study will be achieved through the sequential achievement of the following objectives: (1) recruitment of eligible participants from the Olivet Christadelphian Care Home, Acocks Green, Birmingham, UK. (2) Baseline assessment of the patient-centred outcomes related to multi-dimensional health. (3) Assessment of the feasibility of the study as it relates to the 8 primary areas of focus for feasibility studies [15]. (4) Post-intervention assessment of all feasibility and patient-centred outcomes.

The research questions of this study relate to the eight aforementioned areas of focus of this feasibility study, incorporating the following questions relating to the feasibility and potential efficacy of the study within this setting: can it work? Will it work? Does it work? [15] (Table 1).

**Table 1:** The eight primary areas of focus, outlining the research questions and methods of assessment

Area of focus	Research questions	Methods of assessment
Acceptability	• Will the proposed population be interested in participating in the study? • What will the uptake be? • Will the programme be judged as suitable by the delivers of the programme in addition to the programme participants? • What are participant’s opinions on hypothetically being randomised into a control group during a proposed future clinical trial?* *Participants within the feasibility study will not be recruited as participants within the proposed future clinical trial in order to protect the scientific validity of a future clinical trial, as the participants within the feasibility study will already have undergone the interventions. Additionally, the intervention may be altered after being informed by this feasibility study as well as utilising Patient and Public Involvement (PPI)).	• Participant uptake analysis (all participants approached and eligible for the study/all of those successfully recruited to the study) • Semi-structured interviews with participants • Focus groups with intervention implementers and study support staff.
Demand	• Will the proposed population of care home residents participate in the study? • What will adherence rates be? • Are the staff in the home open to the idea of having an exercise intervention potentially in the home long term if it proves effective?	• Analysis of uptake rates • Exercise intervention adherence rates • Focus groups with study support staff/care home staff.
Implementation	• What are the possible logistical issues with the setting which will need to be addressed or accounted for prior to the clinical trial? • Can the interventions be successfully carried out within this setting? • Can a single or double bind be successfully implemented within the setting?	• Semi-structured interviews with study participants. • More in-depth with focus groups with intervention implementers and study support staff.
Practicality	• What are the practical implications of the study with relation to the time commitment of the researchers, relating to both the implementation of the intervention, and the testing of participants for the dependent variables of the proposed future clinical trial? • Is it viable to potentially conduct follow-up testing on participants in the proposed future clinical trial 12 weeks after the intervention at 24 weeks? • Do any alterations need to be made to the proposed primary dependent variables of the future clinical trial? • If the intervention is successful in influencing parameters of health and functional capacity, will it potentially be possible to assess if these improvements are sustained during a 12-week follow-up in the proposed future clinical trial if the same is found?	• Semi-structured interviews with study participants. • Focus groups with support staff and intervention implementers.
Integration	• How will the care home staff appraise the study? • Will the intervention be easily integrated into the existing culture, protocols and procedures within the care home seamlessly?	• Focus groups with support staff and intervention implementers.
Adaptation	• Will any further adaptations be required to the existing intervention to make it more feasible or appropriate within this setting?	• Focus groups with intervention implementers. • Semi-structured interviews with participants.
Expansion	• Can the HUR equipment be successfully utilised in (and its use expanded to) this setting?	• Semi-structured interviews with study participants • Focus group with intervention implementers.
Limited-efficacy testing	• Is 6 weeks (or potentially 12 weeks in the case of the proposed future clinical trial) a sufficient duration to potentially provide significant benefit to patients? (This will inform the time points at which testing will occur within the future clinical trial) • Can a moderately intensive (3–4 days per week), 6-week (12 weeks potentially in the case of the proposed future clinical trial) specially adapted resistance training intervention improve markers of multi-dimensional health in frail elderly individuals?	• Analysis of the patient-centred outcomes within the study (primary dependent variables of the proposed future clinical trial). • Analysis of uptake and adherence rates. • Analysis of the level of satisfaction with the interventions through semi-structured interviews and focus groups with participants and intervention implementers respectively, post intervention.

### Design overview

**Fig. 1:**
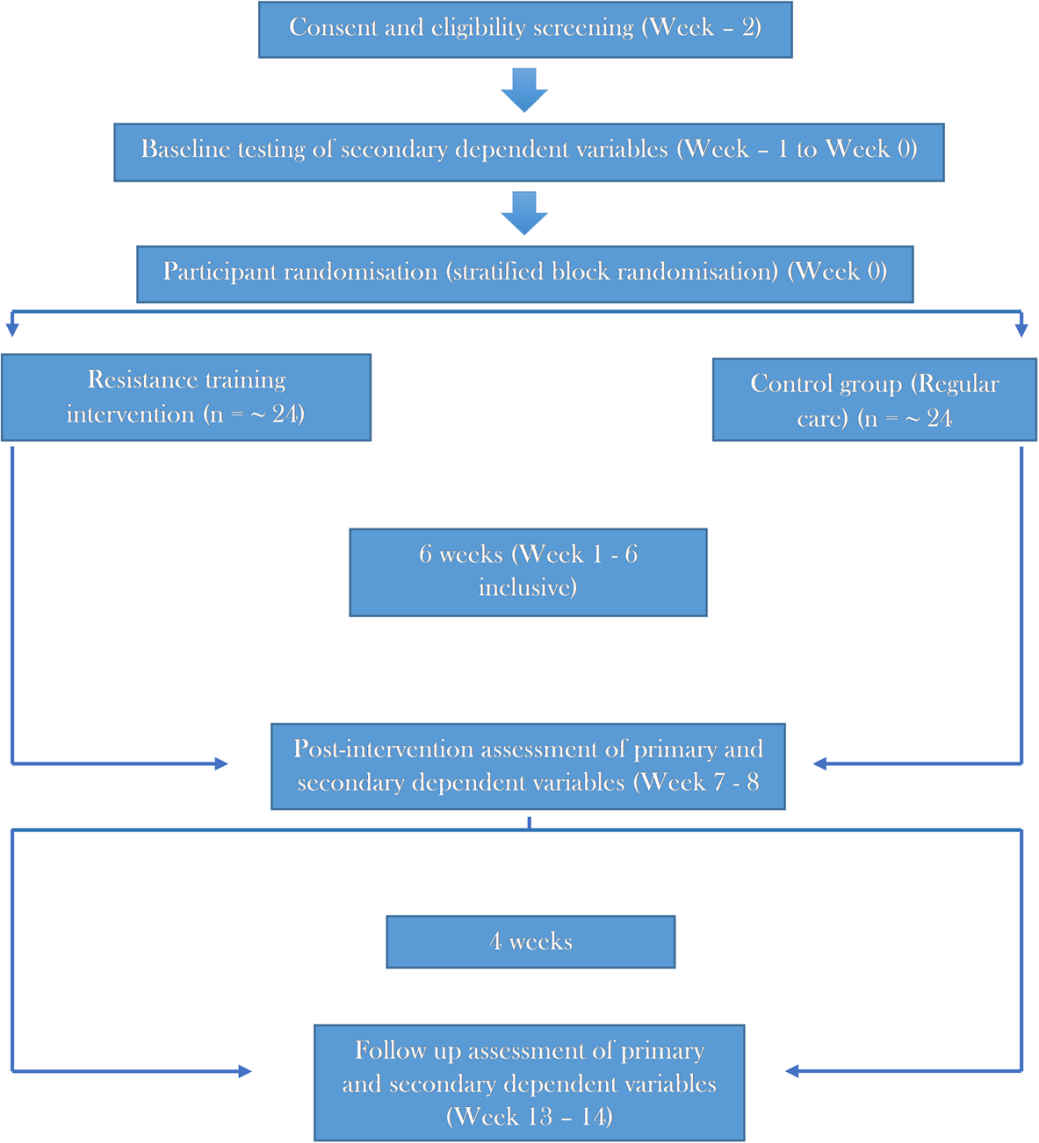
Trial schema of participant flow throughout the duration of the study

This feasibility study will utilise a 12-week, interventional, randomised, and independent measures research design (Fig. 1).

The independent variables of the study will comprise of a specially adapted resistance training intervention, and a control group which will receive regular care. A wait-list control group will be utilised within the proposed future clinical trial, but a concurrent control group will be utilised within this feasibility study.

In order to ensure this present study is as scientifically valid as possible a number of precautions have been taken to protect the internal and external validity of the study within its methodological design. First, for each participant, all testing procedures (baseline, post-intervention (6 weeks) and follow-up (12 weeks)) will be conducted at approximately the same time of day (± 2 h). This will be controlled in order to protect the findings of the study from changes in the patient-centred outcomes which may be attributable to circadian variation rather than manipulation of the independent variable [19]. The hypothesis of the study will not be divulged to participants prior to or during the conduction of the study in order to control for any potential degree of demand characteristics; a scenario where participants alter their behaviour and/or answers, in order to align with what they believe is potentially the ‘desired’ outcome of the study. All testing sessions related to patient-centred outcomes will take place at least 24 h after the cessation of the previous training session for each participant. This will be implemented in order to ensure acute fatigue does not only become a contributing factor to the results of the study, specifically relating to the patient-centred outcomes, but also the feasibility of such practice during a proposed future clinical trial. All participants will also be asked to refrain from any relatively high-intensity exercise training up to 24 h prior to each testing session. Due to an independent measures research design being employed, a control group will be employed in order to increase the internal validity of the study, i.e. increases the likelihood that any potential changes in the patient-centred outcomes of the intervention group are due to the intervention and not additional external factors. The order in which patient-centred outcomes are tested will be counter-balanced throughout the study at each assessment time point in order to attempt to protect the study from practice effects, especially in the form of order effects, where a participant has been exposed to a specific order of testing before and as such performs better on subsequent testing procedures of the same material. Stratified-block randomisation of participants (based on frailty score and age) will also be implemented in order to reduce any differences between participants within each of the independent variable groups at baseline. This will also allow for protection against additional threats to the internal validity of the study, such as the influence of passing time (unrelated to the intervention) on participants within the study (maturation), and also protects against potential subconscious selection bias amongst the research team relating to group allocation of participants. Finally, in order to increase the external validity of the study, the eligibility criteria of this present study will be kept as minimalistic as possible (within the limits of safety and reason), in order to allow as inclusive a proportion of this population as possible, and in such producing findings which are applicable to not only those within the study, but to the greater population of frail geriatric older adults within residential care settings.

### Eligibility

This study is open to both men and woman whom meet the following eligibility criteria: presently, a resident within the Olivet Christadelphian Care Home, Acocks Green, Birmingham, UK; ≥ 65 years of age; frail according to the Fried frailty phenotype criteria [2]; have the capacity to speak and read in English; not currently taking part in any other clinical trial which could potentially impact upon or influence the findings of this present study; not currently terminally ill with life expectancy which is less than the duration of the follow-up of the study; does not have any severe sensory impairment which would profoundly impact upon their capacity to undergo the intervention, even once appropriate adaptations have been made.

### Intervention

**Fig. 2:**
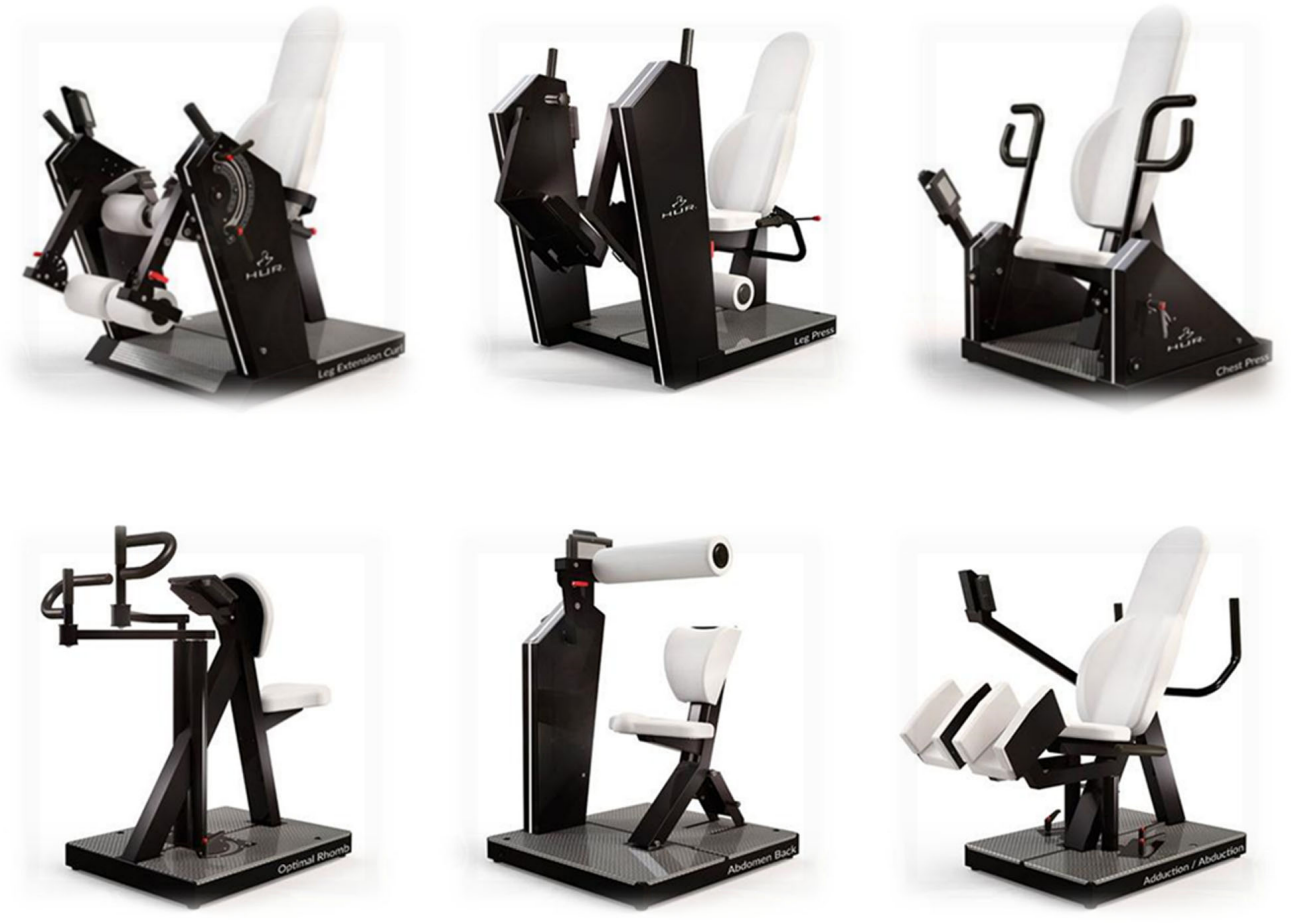
HUR Ltd. resistance training equipment utilised within the interventional arm of the study

The intervention within this study will be comprised of a moderately intensive, 35 min per session, 3–4 sessions per week, 6-week, machine-based resistance training intervention. The sessions will be conducted in groups of approximately six individuals in the form of a group exercise circuit. Study participants will perform exercises predominantly targeting the lower limbs (but also upper limbs and core) on six separate pieces of resistance training equipment: leg extension, leg curl, leg press, chest press, back row, seated abdominal crunch, and thigh abduction/adduction training machines (HUR Ltd., Helsinki) (Fig. 2).

**Fig. 3:**
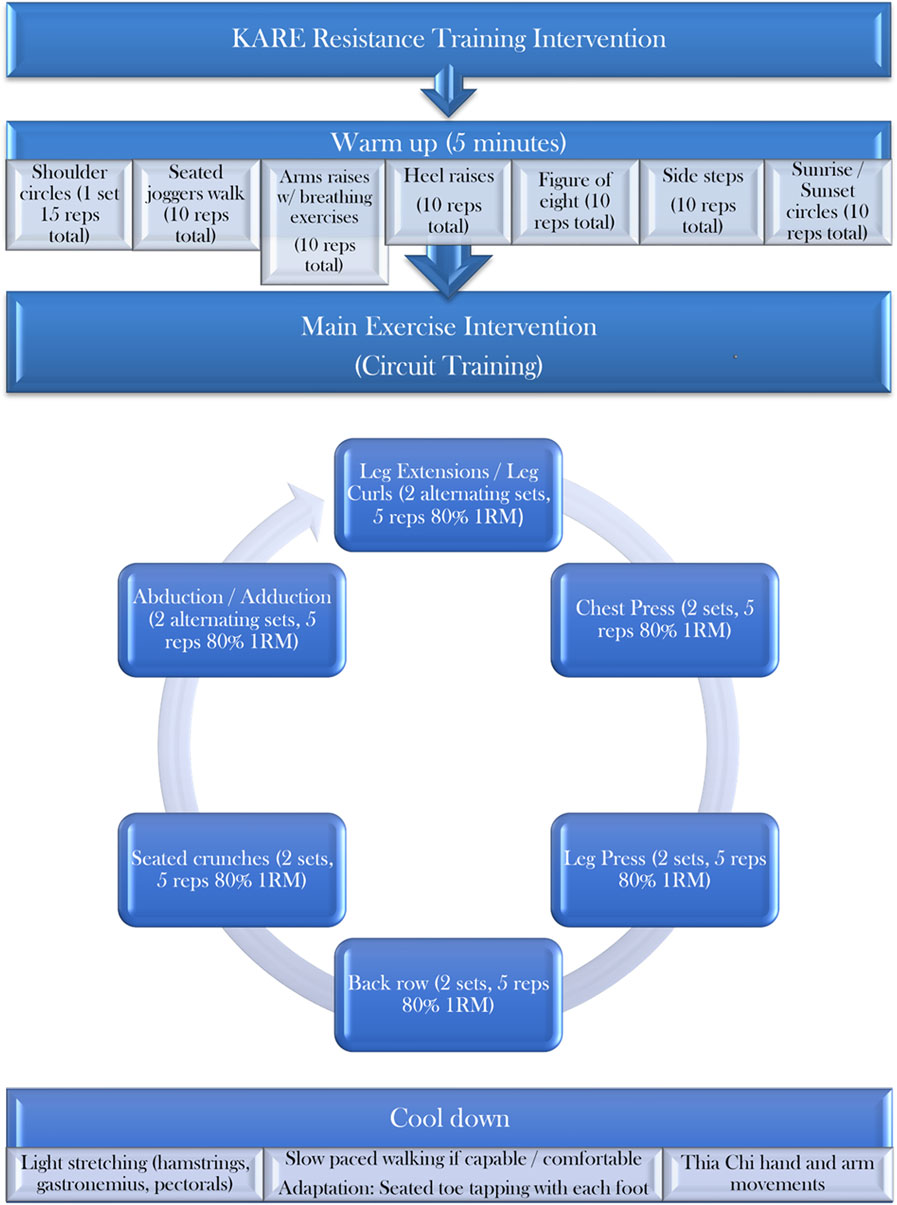
Interventional exercise session protocol

All sessions will be performed under the guidance of a qualified trainer, and all participants will undergo 21 sessions in total throughout the 6-week intervention. An outline of the protocol for each session can be found in Fig. 3.

The intervention will commence with three sessions per week for the first week, followed by four sessions per week for the second week. This pattern will alternate throughout the duration of the study (Table 2). A maximum of one session will be performed each day, and sessions will not be performed on any more than a maximum of two consecutive days throughout the duration of the study to reduce fatigue and the risk of delayed onset muscle soreness or injury. As previously mentioned, sessions within the resistance training intervention group can facilitate a maximum of six participants per session (limited by the number of resistance training machines). As such dependent on recruitment numbers, the intervention will be conducted in multiple different sessions during each training day and separated into different groups if necessary. These individual groups will not be compared to one another.

**Table 2:** Study timeline of all major events throughout the duration of the study (SPIRIT Schedule)

Week	Keeping Active in Residential Elderly (KARE) study timeline						
	Monday	Tuesday	Wednesday	Thursday	Friday	Saturday	Sunday
Week - 2	Consent and eligibility screening	Consent and eligibility screening	Consent and eligibility screening	Consent and eligibility screening	Consent and eligibility screening	Consent and eligibility screening	–
Week - 1	Pre-intervention assessments	Pre-intervention assessments	Pre-intervention assessments	Pre-intervention assessments	Pre-intervention assessments	Pre-intervention assessments	Rest
Week 0	Pre-intervention assessments	Pre-intervention assessments	Pre-intervention assessments	Pre-intervention assessments	Pre-intervention assessments	Pre-intervention assessments	Rest
Week 1	Training	Rest	Training	Rest	Training	Rest	Rest
Week 2	Training	Rest	Training	Rest	Training	Training	Rest
Week 3	Training	Rest	Training	Rest	Training	Rest	Rest
Week 4	Training	Rest	Training	Rest	Training	Training	Rest
Week 5	Training	Rest	Training	Rest	Training	Rest	Rest
Week 6	Training	Rest	Training	Rest	Training	Training	Rest
Week 7	Post-intervention assessment	Post-intervention assessment	Post-intervention assessment	Post-intervention assessment	Post-intervention assessment	Post-intervention assessment	–
Week 8	Post-intervention assessment	Post-intervention assessment	Post-intervention assessment	Post-intervention assessment	Post-intervention assessment	Post-intervention assessment	–
Week 9–12	Participants within the resistance training intervention group will have access to the machines but no formal exercise programme will be in place. (All activity within this period will be automatically recorded on an electronic database for each participant)						
Week 13	Follow-up assessment	Follow-up assessment	Follow-up assessment	Follow-up assessment	Follow-up assessment	Follow-up assessment	–
Week 14	Follow-up assessment	Follow-up assessment	Follow-up assessment	Follow-up assessment	Follow-up assessment	Post-intervention assessment	–

Additionally, all participants within the intervention will have continual access to the resistance training equipment between the end of the post-intervention assessments, and the follow-up assessments. Between the post-intervention assessments and follow-up assessments, participants and care staff will be encouraged to have participants utilise the machines as much as possible during this period, and although no formal exercise programme will be in place, participants will have access to the machines and the session protocol previously utilised. The unique aspect of these machines is that they require participants to scan an ID card prior to use and all user data (the number of repetitions, sets and the loads lifted) is stored on an electronic database accessible to the researcher at any time. As such, this will provide interesting feasibility data relating to the continued use of the equipment after the formal study-based intervention has concluded.

### Outcome measures

#### Feasibility outcomes

The feasibility outcomes of this study will relate to the eight primary areas of focus of feasibility studies [15] (utilised to establish the feasibility of a proposed future clinical trial within this setting), relating to acceptability, demand, implementation, practicality, adaptation, integration expansion and limited-efficacy testing.

These feasibility outcomes will be assessed through semi-structured interviews with study participants and focus with intervention implementers and study support staff post-intervention. Participant uptake and adherence records will also be employed throughout, as will questionnaires with study participants, intervention implementers, and study support staff. These methods will seek to attain answers to the following questions and parameters relating to the eight primary areas of enquiry for this feasibility study outlined in further detail in Table 1.

In order to enhance trustworthiness in the qualitative component of this research, several methods will be employed**:**

The researcher gathering the data will keep a reflective journal in which they will record information about themselves, their activities and the methods used. Field notes will include time, date and location, participant’s actual notes, the researcher’s own questions and comments. This will lend to logging and documenting what is learned about the study, the intervention, the setting, the participants, and used to refine focus for future interviews through assessing the following questions: what is important? What is it I need to find out more about? What would I want to focus on more closely if I could do the interview again, or in future interviews? [20].

Data will be gathered from study participants, study support staff and intervention implementers in order to collect data from multiple sources (triangulate information). This study will also employ more than one researcher to analyse the qualitative data in order to enhance triangulation and validity.

Enough details will be given about the participants and the setting to make decisions about the quality of the findings from the qualitative analysis. Detailed descriptions about the participants’ experiences and the setting will be provided by the researcher.

In the qualitative data analysis, clarification of all possible researcher biases will be made known. For example, it will be articulated that the researcher is an advocate of physical activity as a means to promote health, prescribing to the theoretical and practical concept of exercise as medicine, and hence there may be some form of unconscious subjective bias in this context. However, it should also be noted that the researcher within this study is also an advocate of science to an equal or even greater extent, and as such any such bias in subjective analysis would potentially be counteracted in this sense.

Interviews—“a conversation with a purpose” [21] will be the primary method of data gathering utilised within this feasibility study, as it enables large amounts of information to be gathered relatively quickly. Specifically, this study will employ semi-structured interviews, with open questions in a conversational format. There will be a number of pre-determined themes, topics and questions to be discussed, specifically relating to the eight areas of focus of the feasibility outcomes of this study. All interviews will be audio-recorded in order to facilitate future transcription. This will also be the case with focus groups with study support staff and intervention implementers. The qualitative element of this study will also explore opportunities for Patient and Public Involvement (PPI) in the research design of the proposed future clinical trial.

As this study will utilise a mixed-methods research approach, employing both qualitative (feasibility outcomes) and quantitative (patient-centred outcomes) research methods, this will provide researchers with the opportunity of not only gathering the individual data needed from each method of data collection, but will also facilitate the potential for elaboration and expansion of these findings through complementary analysis of each approach. The qualitative aspect of this feasibility study, aimed at assessing the feasibility outcomes, will predominantly take a phenomenological approach to understand the experiences of individuals involved in the study [22].

#### Patient-centred outcomes

The patient-centred outcomes of this feasibility study relating to multi-dimensional health (and comprising the proposed primary dependent variables of the future clinical trial) are as follows:

Physiological*: Serum Cortisol, Dehydroepiandrosterone (DHEAS), cortisol: DHEAS ratio, C-reactive proteins (CRP), Interleukin 6 (IL-6), Tumour Necrosis Factor alpha (TNFα), Interferon gamma (IFNy).

Functional: Hand grip strength (Southampton protocol [23]), leg strength and power output [24, 25], Short Physical Performance Battery (SPPB) [26], Katz Index of Independence in Activities of Daily Living (Katz ADL) [27], Fried Frailty Phenotype [2].

Psychological/Emotional: Geriatric Depression Scale (GDS) [28], Hospital Anxiety Depression Scale (HADS) [29], Perceived Stress Scale (PSS) [30].

Cognitive: Standardised Mini-Mental State Examination (SMMSE) [31].

Social: Interpersonal Support Evaluation List (ISEL-12) [32].

*All blood samples will be obtained through the process of phlebotomy (venepuncture). Serum will be analysed for the physiological patient-centred outcomes relating to cortisol and DHEAS (assessed by commercial ELISA kit). C-reactive protein and inflammatory cytokines (IL-6, TNFα, IFNy) will be assayed using a multiplex commercial kit (R&D Systems).

#### Identification, consent and recruitment

##### Identification

To identify potential participants, residents will initially be screened by their care team for the following criteria: aged ≥ 65, no of severe sensory impairments that would profoundly impact upon their ability to participate, able to speak and read the English language, not currently taking part in any other clinical trial which could potentially affect the results of this current study, and with a life expectancy which is greater than the length of the study. Potential participants will first be approached by a member of their regular care team at the care home with an information sheet related to the study and asked if they would either be interested in participating in the study or if they would like to receive more information. The information sheets will contain all of the most pertinent information relating to the study and in particular what it would require from potential participants. Potential participants will be given approximately 1 week, after receipt of the information sheet, to consider whether or not they would like to participate in the study. If potential participants express their interest in the study, a member of the research team will meet with them to provide them with more information on the study, and to address any queries which they may have (Additional file 1).

##### Consent

At this stage, potential participants will also be provided with an informed consent form and asked if they would be interested in participating. If it is deemed that a potential participant lacks the capacity to consent, a personal consultee will be sought. If a personal consultee cannot be found, then a nominated consultee will be sought. All efforts possible will be made in this regard to include participants who lack the capacity to consent within the study, as intrinsically within the research team from a personal and professional perspective we would consider it unethical to exclude potential participants from participating in a study, which can potentially benefit them and their overall health status, simply due to the fact that they lack the capacity to consent.

##### Recruitment

Following consent being obtained from the participant themselves, or the obtainment of a declaration from a consultee, all consented potential participants will be screened for the remaining eligibility criteria relating to frailty status.

#### Randomisation and concealment

To restrict the chances of imbalance between the intervention and control groups within this present study (in addition to the proposed future clinical trial) a stratified block randomisation strategy will be employed [34], in order to achieve balance relating to participant baseline characteristics (covariates) for frailty score and age [35]. This randomisation procedure will be carried out through a computer-generated programme [36], by a competent staff member, otherwise unrelated to the study, and the project as a whole. Allocation concealment will be employed where the researcher responsible for recruiting participants and gathering data from the participants will be unaware of the group to which each participant will be allocated initially until initial data collection is complete, avoiding both conscious and subconscious selection bias [37–39].

#### Data collection

Data within this feasibility study will predominantly be collected at three main time points: baseline, post-intervention, and follow-up (Table 2).

##### Baseline assessment

Participant’s baseline socio-demographic and information for the patient-centred outcomes of the study will be collected between 12 days and 36 h prior to the commencement of the 6-week intervention. One repetition maximum (1RM) for all of the resistance training equipment utilised within the resistance training intervention will also be assessed during this time period (after all baseline testing has been completed, and at least 18 h after baseline testing which requires physical exertion, which may impact on the accuracy of the 1RM measurements).

##### Post-intervention assessment

The feasibility and patient-centred outcomes of the study will be assessed between 10 h and 12 days post the cessation of the 6-week intervention. All assessments will take place at least 24 h post the cessation of the last exercise training session.

##### Follow-up testing

Follow-up testing will be conducted 6 weeks post-intervention cessation for the feasibility and patient-centred outcomes of the study. All data will be gathered within a period of approximately 12 days from all participants.

Adherence rates in the intervention group will be recorded as the number of repetitions completed in a set (90% required for adherence to that exercise), and then the number of exercises for which there was a 90% adherence. If participants meet these parameters for each exercise session, they will be considered to be in 100% adherence to the intervention. For example, if a participant performs 95% of all exercises in one session, then they will be considered to be in adherence for that session. If they then continue this level of adherence for the remaining 41 sessions, they will have a 100% adherence. If a participant adheres to 95% of the intervention for 36 sessions, but only 80% for 6 sessions, then they will have an 86% adherence rate. Ninety percent of all exercises performed within that session will signify adherence to that session. Adherence rates, whether very high or somewhat low may signify that the intervention may have been too demanding, too easy or optimal. Information will also be collected throughout the study related to uptake and retention rates.

#### Data monitoring

Data will be monitored by the trial management committee at monthly intervals. Prior to analysis, data entry checking will be conducted for accuracy on 10% of all participants, and queries will be resolved through discussion with the trial management committee and access to the source documents held at the university. Data management will adhere to the PANINI data management plan, which was developed in accordance with national and European principles as part of the university research governance and European Commission research governance principles. Thus, data management for this project adheres to the FAIR principles [33].

#### Sample size

This study aims to recruit a convenience sample of *n* = ~ 48 participants: 24 intervention and 24 regular care control. No formal power calculations were conducted due to the feasibility nature of this study. This estimated sample size is based on optimistic projections following preliminary discussion with care home residents with relation to potential uptake.

### Statistical methods

#### Qualitative analysis

Analysis of the feasibility outcomes of this study will be based on an inductive process, which utilises Interpretative Phenomenological Analysis (thematic analysis). Two researchers will be employed to analyse the data acquired in order to increase triangulation from the analysis perspective, having already triangulated data acquisition through data obtainment from multiple sources (i.e. study participants, intervention implementers and study support staff). All semi-structured interviews and focus groups will be audio-recorded. Data synthesis will be performed through verbatim transcription of the semi-structured interviews and focus groups. The three main steps of Interpretative Phenomenological Analysis will be followed [22]: (1) the generation of themes from transcripts within the areas of feasibility inquiry. As an iterative process, these themes will be continuously reviewed and adapted based on the emergence of information in subsequent transcripts. (2) The collation and separation of these themes within each of the areas of feasibility inquiry. (3) Written interpretation of the resultant themes within each of the areas of feasibility and their relationship to one another. At all stages within this process, reflective journal entries and field notes will be utilised to provide a more comprehensive understanding of the findings, in addition to incorporating additional feasibility information related to uptake and retention rates, and limited-efficacy testing of the patient-centred outcomes in the final analysis to provide a comprehensive assessment of the feasibility of the study.

#### Quantitative analysis

Statistical analysis of the patient-centred outcomes will be performed using IBM SPSS (Statistical Package for Social Sciences) software. These analyses will be performed as part of the limited-efficacy testing regarding the potential impact of the intervention on the patient-centred outcomes (proposed primary dependent variables of the future clinical trial). Specifically, for this research, the type of statistical analysis which will be used will be as follows: 2 × 3-way independent measures ANOVA’s (analysis of variance consisting of a two independent variables; the specialised resistance training intervention, and control group, each with three levels: baseline, post-intervention (6 weeks) and follow-up (12 weeks) will be carried out for all patient-centred outcomes. A subsequent post-hoc test will be utilised if a significant main effect or interactions are found. Pearson product correlations will also be utilised between various socio-demographic variables (such as age and sex) and the patient-centred outcomes of this study to assess possible relationships between differences in these socio-demographic factors and changes in the patient-centred outcomes of the study.

Central tendency and variability measurements consisting of the measurement of parameters such as the mean, median and mode, and standard deviation and range of scores respectively, will also be utilised during the analysis of data for illustrative purpose. Significance levels will be set at 0.05 (*p* ≤ 0.05), and effect sizes will be reported for all analyses. Additionally, in order to establish if the assumptions of parametric statistics have been met in relation to the assumption that there is a normal distribution of data, the data will be analysed for skewness and kurtosis. As the quantitative component of this study has not been powered given the feasibility nature of the study, the examination of the efficacy of the intervention to impact these variables is limited and interpretation treated with caution pending the results from the future powered clinical trial. All results will be reported with 95% confidence intervals.

### Data storage and protection

Participants’ identity or other personal information will be kept confidential. Participants will be assigned a unique ID number under which all study information will be stored in a secure file and saved on an encrypted and password-protected computer and laptop at the University of Birmingham (UoB). Physical data (e.g. case report forms (CRFs)) will be identifiable only by an ID number and stored in a locked filing cabinet at the School of Sport, Exercise and Rehabilitation Sciences at the University of Birmingham, accessible only by the research team. Participants’ personal data (name and date of birth) and consent forms matching them to their ID number will be stored securely in a locked filing cabinet, separate from all other data and/or in a password protected master sheet on an encrypted and password-protected computer and laptop at the University of Birmingham.

All serum samples will be stored in Human Tissue Act (HTA) complaint facilities at the University of Birmingham for up to 3 years then destroyed. Anonymised whole blood samples will be transferred to the University of Bologna for DNA methylation analysis on candidate genes related to nutrition and physical activity effects on the ageing process as part of an already ethically approved study which is part of the PANINI network, then destroyed at the end of the PANINI trial in Bologna (end of 2019).

All hard copy data collected on CRFs will be stored in a linked-anonymised format securely for 10 years then destroyed. All personal data (consent forms, master sheet linking participant IDs to names and contact details) will be stored for 10 years then destroyed. All computerised data will be archived on UOB servers in anonymised form for 10 years in the first instance in accordance with the UoB Code of Practice for Research, and the Data Protection Act (1998).

Following analysis for this specific study, all data will be anonymised and also entered into a European ‘PANINI’ open access database that this project is part of and optionally may be analysed in future ethically approved research across the PANINI network. The PANINI shared dataset will be made open access at the conclusion of the funding for the PANINI network including this study in 2020 and stored for at least 10 years as an open-access searchable published dataset.

## Discussion

### Implication of the research

As this is a feasibility study, the most immediate implication from a research prospective is the assessment of the feasibility of the proposed future clinical trial within this setting; which will allow for a more detailed, informative and robust understanding of the influences of the specially adapted resistance training intervention on the primary dependent variables of the future clinical trial (the multi-dimensional health and functional capacity of frail older adults within residential care settings). Additionally, frailty can also have an enormous impact on an individual’s life, in addition to the lives of their loved ones, and even an impact on society as a whole [10]. As such, if the study does prove feasible, and the limited-efficacy testing proves positive, this study also has the potential to have far reaching implications; most importantly leading to the advancement of care for frail geriatric populations within residential care settings and the ability to measurably improve various aspects of their overall health and functional ability, as well as benefitting the lives of their loved ones.

### Dissemination

The findings of this study will be disseminated through publication in the form of scientific papers in open access scientific journals, public engagement events within the UK and Europe (a core element of the PANINI project’s aims and objectives), online via social media (Twitter, Instagram) and the PANINI project website (40), presentation at various conferences within the UK, Europe and the rest of the world, and to study participants upon request, as they become available.

### Safety reporting and monitoring

Adverse events (AE) and serious adverse events (SAE) will be monitored and recorded. AE will be reviewed, while SAE will be reported immediately through completion of a SAE form indicating causality and severity (in liaison with an appropriate expert) and submitted to the study sponsor and REC within 24 h. SAE related to pre-existing conditions will not be reported. Standard actions following an AE or SAE would be a referral to a general practitioner or accident and emergency services, and to recommend that the participant withdraw from the study unless they have been cleared to continue exercise by their attending physician.

### Trial registration

This study has been registered on ClinicalTrials.gov under the identifier number NCT03141879.

### Trial status

This trial has received ethical approval and is due to be conducted in 2019 at the Olivet Care Home, Sherbourne Road, Acocks Green, Birmingham, with completion of data collection scheduled prior to 01 December 2019.

## Additional file

Additional file 1: Keeping Active in Residential Elderly (KARE) participant information sheet. (DOCX 1880 kb)

## Data Availability

Not applicable.

## Abbreviations

1RM: One repetition maximum
ADL: Activities of daily living
AE: Adverse events
ANOVA: Analysis of variance
CRF: Case report form
CRP: C-reactive proteins
DHEAS: Dehydroepiandrosterone
ELISA: Enzyme-linked immunosorbent assay
GDS: Geriatric Depression Scale
HADS: Hospital Anxiety and Depression Scale
HTA: Human Tissue Act
IFNy: Interferon gamma
IL-6: Interleukin-6
IPA: Interpretive Phenomenological Analysis;
ISEL-12: Interpersonal Support Evaluation List-12
KARE: Keeping Active in Residential Elderly
NHS: National Health Service
PANINI: Physical Activity and Nutritional INfluences In ageing
PI: Principal investigator
PPI: Patient and Public Involvement
PSS: Perceived Stress Scale
REC: Research Ethics Committee
SAE: Serious adverse events
SMMSE: Standardised Mini-Mental State Examination
SPPB: Short physical performance battery
SPSS: Statistical Package for Social Sciences
TNFα: Tumour necrosis factor alpha
