# Supplementary material for: Assessing the feasibility and impact of an adapted resistance training intervention, aimed at improving the multi-dimensional health and functional capacity of frail older adults in residential care settings: protocol for a feasibility study
Source: Pilot Feasibility Stud. 2019 Jul 5;5:86. doi: 10.1186/s40814-019-0470-1 (PMC6612216; doi:10.1186/s40814-019-0470-1)
Supplement: Supplementary file 1 — Keeping Active in Residential Elderly (KARE) participant information sheet. (DOCX 1880 kb) [file 40814_2019_470_MOESM1_ESM.docx]

**Keeping Active in Residential Elderly (KARE)**

**Participant Information Sheet**

1. **What is this study about?**

This research study seeks to assess whether it is feasible to conduct a specialised, seated physical activity intervention for frail older adults in a residential care setting. The intervention will run three – four times per week (35 minutes per session) for six weeks, with a further six-week follow-up. We are interested in the effects on your health and wellbeing.

The specialised chair-based physical activity intervention will consist of specially adapted and designed resistance training machines for frail older adults. A Computer Aided Design (CAD) of the intervention set up in the care home is displayed on the final page of this information sheet (Figure 1). The six specially adapted physical activity machines which will be used in the intervention are also displayed on the last page (Figure 2).

**HUR Physical Activity Intervention**

-
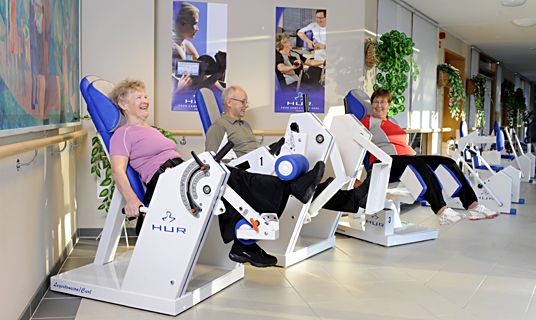
The HUR physical activity machines are specially adapted resistance training machines for older adults.
- They are comfortable, easy to use, and are pneumatic (work on air-based resistance – so there are no weight plates involved).
-
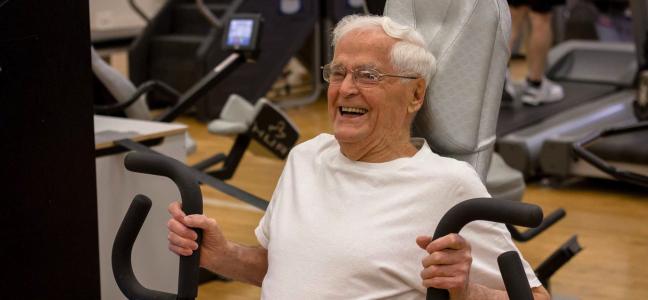
They allow the difficulty to be increased in small amounts (100 grams - which is similar to the weight of a small apple).
- The machines work by scanning your wristband on a touch screen, and pressing buttons to increase or decrease difficulty.
- It will also record all your activities and store them for your next session.

1. **Who is organising and conducting this study?**

The Physical Activity and Nutritional INfluences In ageing (PANINI) project research group at the University of Birmingham.

Departments Involved: School of Sport, Exercise and Rehabilitation Sciences, University of Birmingham.

Chief Investigator: Professor Anna Whittaker ([a.c.whittaker@bham.ac.uk](mailto:a.c.whittaker@bham.ac.uk) / 0121 414 4121)

Research Team: Mr. Paul Doody ([p.d.doody@bham.ac.uk](mailto:p.d.doody@bham.ac.uk) / 0121 414 4125)

Professor Janet Lord ([j.m.lord@bham.ac.uk](mailto:j.m.lord@bham.ac.uk) / 0121 371 3234)

1. **Why have I been approached?**

The care home has been chosen as the research site for this feasibility study, having been visited multiple times by the research team and talking to residents, we believe this is an ideal location for the study, the purpose of which is primarily to inform the feasibility of a proposed future clinical trial within this setting.

In order to be eligible for the study, you must be:

- A resident within the care home.
- ≥ 65 years old,
- Have the capacity to speak and read in English,
- Not currently taking part in any other research study which could affect the findings of this present study
- Frail (Possessing any three of the following criteria):
- Low levels of handgrip strength,
- Unintentional weight loss,
- Low levels of physical activity,
- Slow walking speed, and
- Self-reported exhaustion

*If you would like to participate but are unsure if you are eligible based on the criteria above, do not worry, as these criteria will be assessed by the research team prior to enrolment once you have expressed your interest.


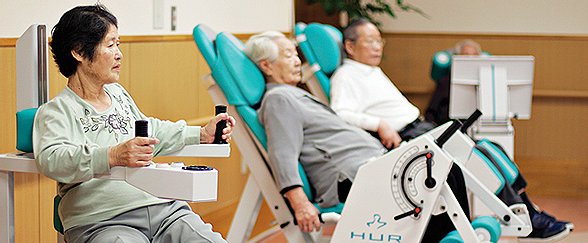


1. **What will happen to me if I take part?**

Your participation will be up to 4 months including testing before and after and the physical activity intervention itself. There will be no other changes to your standard care.

**Before the intervention:**

**Screening process:** Initially you will be asked to undergo an eligibility screening related to the criteria mentioned on the previous page, between 21 – 14 days prior to the intervention. This will involve a series of short tests, and should take a total of about one hour, at which point we will be able to inform you whether or not you are eligible for the study.

**Baseline Assessments:** If you are eligible, up to 14 days prior to the intervention you will be asked to undergo a series of measurements relating to your physical, psychological, cognitive (will be assessed at the end of the eligibility screening session up to 21 days prior), emotional and social health and physical ability. These will be assessed through tests of function, questionnaires, and a blood sample to measure immune function. These will all together take about five hours in total and will be divided into three sessions over the 12 days. The first two sessions will last approximately 1 hour 30 min in duration. The third session, where you will become familiarised with the equipment and we will also assess the weight you will be using on the machines, will last about 2 hours in total, with lots of rest if you think you will need it. Although we would ideally like you to complete all of the assessments, during the first two assessments, if you feel it is too much, we will prioritise certain tests.

**During the intervention:**

You will then be randomised into either the HUR resistance training group or a control group which will receive regular care throughout the duration of the study, but who will also be offered the intervention once the follow-up testing of the study has been completed. Randomisation means that you will be allocated by chance to either one of these groups for the duration of the study. If you are randomised into the physical activity intervention group, you will be asked to complete 3 - 4 training sessions per week (41 in total) throughout the six weeks. Each training session will last approximately 35 minutes and will be conducted in groups. All sessions will take place in the care home.

**After the Intervention:**

After the six weeks, for both the intervention and control group, you will be asked to complete the same measurements as before in order to assess the impact of the intervention which will be split into 2 sessions each about 1 hour 30 mins each. You will also take part in an audio-recorded interview (20 mins) with the researcher to help us plan a potential future clinical trial. During this you will be able to tell us your opinion of the intervention / control, what you enjoyed about the study thus far, and where you believe it could potentially be improved, which will allow us to adapt the intervention, and the study based on analysis of your feedback, which we would greatly appreciate. The researchers within this study will also request limited access to your medical notes to allow the research team to assess any factors (injuries, ailments, diseases) which might affect the results of the study. As with all information obtained within the study, information from your patient notes will be stored confidentially and separately from your personal details.

**Follow-up**

After the post intervention assessments at week 6, participants in the intervention group will have free use access to the resistance training equipment, although no structured training plan will be put in place. The great thing about the machines is that they will allow us to record exactly how much the physical activity equipment used during this six-week period. At week-12, both groups will be asked to complete the same measurements as before in order to assess if any potential benefits lasted. This will be 3 sessions of about 1 hour 30 mins each. You will also be asked to again take part in an audio-recorded follow-up interview (10 mins) with the researcher, to help us understand your opinion on the previous six-weeks of the study and the follow up testing.

1. **Do I have to take part?**

Involvement is purely voluntary, and you are free to withdraw from the study at any point without giving an explanation. There will be no negative consequences of withdrawing from the study in terms of your care. If you withdraw, we would like to still analyse the data we have collected thus far.

If residents express their interest in the study, a member of the research team will then meet with them to provide them with more information on the study, and to address any queries which they may have. If the care home resident states that they would not like to be involved in the research and would also not like to be contacted in the future with relation to participation, then they will not be approached by the researchers with additional information relating to the study or asked to participate.

1. **What are the possible benefits of taking part?**

Physical activity has been shown to produce positive health benefits in older people. Additionally, physical activity interventions have been suggested as potentially offering the best form of treatment for frail older adults. If this feasibility study proves practical you may also have the opportunity to take part in a future study, testing a 12 weeks physical activity intervention. Participants randomised into the control group, will also be offered the six-week physical activity intervention received by the intervention group, once the study has ended (i.e. after the follow-up assessments of the study are complete). Participants both within the intervention and control may also benefit from a multi-dimensional health assessment at each of the testing sessions.

1. **What are the possible risks/side-effects of taking part?**

Due to the nature of the interventions (physical activity), it is possible that you may experience mild muscle soreness associated with physical activity, however this is a normal adaptation to exercise. In order to minimise the risk of Delayed Onset Muscle Soreness (DOMS – a more severe form of muscle soreness with a delayed onset), the interventions within the study have been developed in a progressive manner. Additionally, the interventions will be carried out in the care home; with care staff in close proximity at all times.

1. **Confidentiality and data protection**

Your identity or other personal information will be kept confidential. You will be assigned an ID number under which all study information will be stored in a secure file, and saved on an encrypted and password protected computer and laptop at the University of Birmingham. Physical data (e.g. questionnaires) will be identifiable only by ID number and stored in a locked filing cabinet at the University of Birmingham, School of Sport, Exercise & Rehabilitation Sciences, accessible only by the research team. Your personal information (name, D.O.B.) and your consent form matching you to your ID number, will be stored securely in a locked filing cabinet, separate from all other data in a password protected master sheet on an encrypted and password protected computer and laptop at the University of Birmingham. However, confidentiality of information provided can only be protected within the limitations of the law. It is possible for data to be subject to subpoena, freedom of information claim or mandated reporting by some profession. It is also obligatory to breach confidentiality in the event that information comes to light that might indicate harm to yourself or other individuals. In the event that we discover certain parameters of tests relating to depression, anxiety, stress or some blood tests that are “unusual”, we will contact you and request your permission to inform your care team of these results, in an effort to potentially to improve your care. None of these tests form a medical diagnosis of any kind, and we will only contact your care team about this with your specific permission to do so. Particularly in relation to blood tests, some tests may be elevated for a wide variety of situations and conditions and it may be that for some people this is a known and acceptable level and is no cause for concern. Data will be stored securely and confidentially for 10 years and anonymised data will also be entered into a European ‘PANINI’ database that this project is part of. Blood samples will be stored in approved facilities at the University of Birmingham. Blood samples may be stored for up to three years for analysis in future ethically approved research across the PANINI network. The University of Birmingham has public liability insurance covering its research. The audio-recordings of all interviews will also be transcribed, and transcriptions stored by ID number only, and the original recordings destroyed in order to further protect anonymity and confidentiality.

1. **Funding**

This study has been funded by the European Commission as part of a European – wide innovate training network (ITN), consisting of Marie Curie Research fellows.

1. **What next?**

If you express that you are interested in taking part in the study to a member of your care team, the research team will come to answer any questions you may have, check you are potentially eligible and enrol you into the study. If you do not express that you are either interested / not interested in the study, the research team will be around to ask you about whether or not you would be potentially interested in the study, and to address any questions or queries you may have. If you would not like to take part, then please express this to your care team and/or the research team and you will not be contacted further in relation to participation.

If you are interested in the study, and agree to take part, we will then ask you to sign an informed consent form, outlining that you have read this information sheet and that you are aware of what the study involves. After this, you will be formally assessed for the eligibility criteria, and then begin the baseline testing measures as soon as possible and then, if you have been randomised into the intervention group, proceed to starting the intervention.

If you have concerns about this study please contact the study research team or your care team to discuss this. For independent advice, you can contact your local Patient Advice and Liaison Service (PALS) or the study sponsor (the University of Birmingham), who has given this study the sponsor registration number: RG_17-108, which should be quoted upon contact:

**Dr. Sean Jennings, Head of Research Ethics and Governance, University of Birmingham, Edgbaston, Birmingham, United Kingdom. Telephone: 0121 415 8011, Email:** [**s.jennings@bham.ac.uk**](mailto:s.jennings@bham.ac.uk)


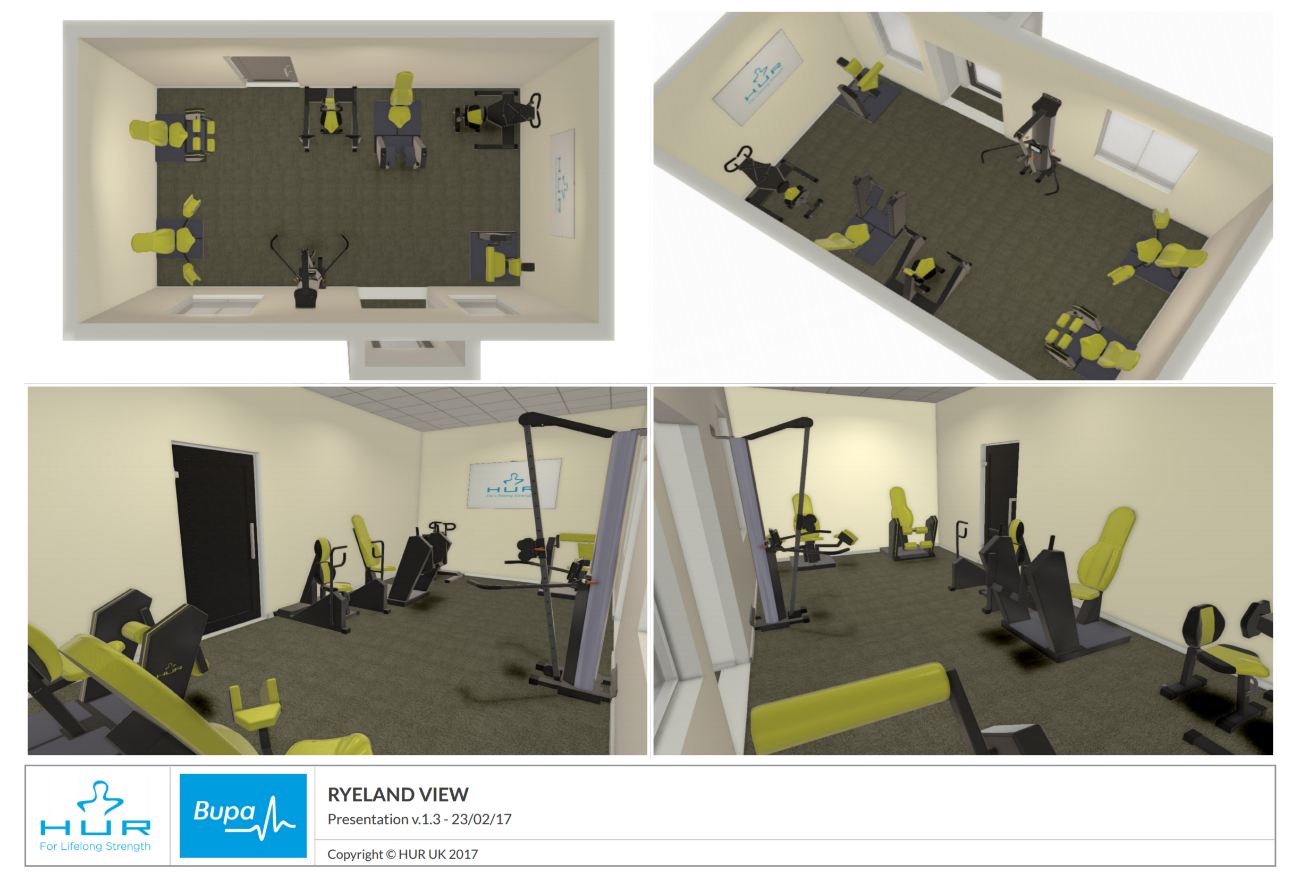


Figure 1. Computer Aided Design of equipment in the room which will be used for the

HUR resistance training intervention within the care home.


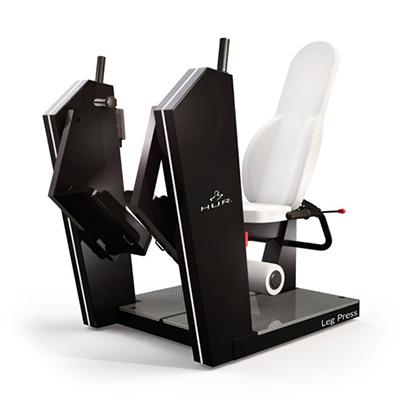

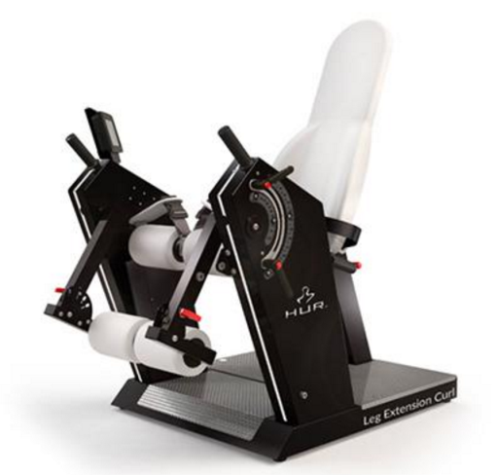

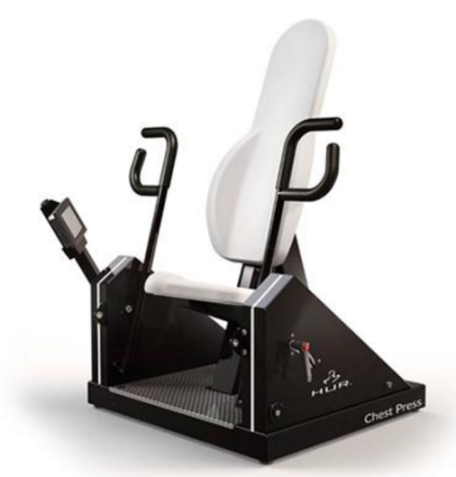


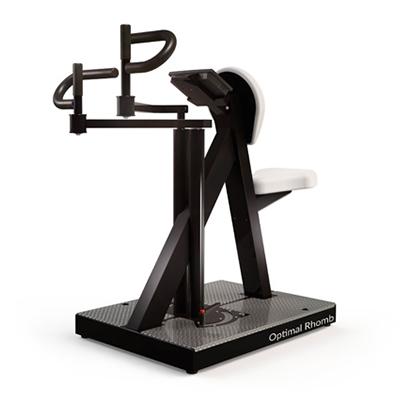

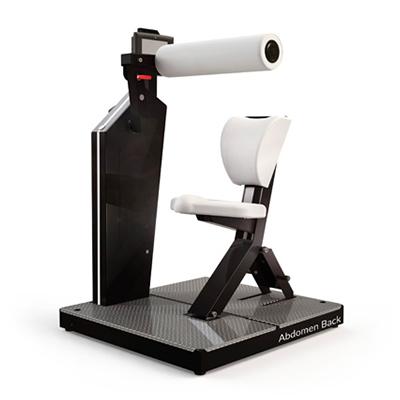

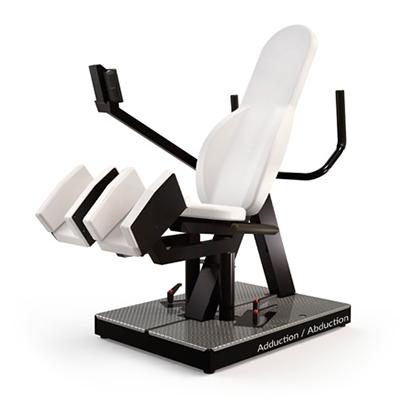


*Figure 2. The HUR equipment utilised within the intervention of the study.*
